# Individual and Social Predictors of Prosocial Behavior among Chinese Adolescents in Hong Kong

**DOI:** 10.3389/fped.2015.00039

**Published:** 2015-05-15

**Authors:** Frank H. Y. Lai, Andrew M. H. Siu, Daniel T. L. Shek

**Affiliations:** ^1^Department of Rehabilitation Sciences, The Hong Kong Polytechnic University, Hong Kong, China; ^2^Department of Applied Social Sciences, The Hong Kong Polytechnic University, Hong Kong, China

**Keywords:** prosocial, predictor, adolescent, Chinese

## Abstract

Based on the human ecological model, this study hypothesized that individual competence in empathy, prosocial moral reasoning, and social influence from parents, peers, and school are the key determinants of prosocial behavior among Chinese adolescents in Hong Kong. We recruited a sample of high school students who engaged in volunteering activities regularly (*N* = 580). They completed a self-administrated questionnaire designed to measure prosocial behavior and its hypothesized predictors using a number of standardized instruments. The results of multiple regression show that social influence factors, including peer, school, and parent influence, are strong predictors of helping intention and prosocial behavior, while individual competence factors like empathy and prosocial moral reasoning are not. Male participants had higher empathy scores and helping intention than females, perceived their parents as more helpful, and their schools as more supportive of prosocial behavior. However, the significant predictors of prosocial behavior and helping intention were similar across gender. The findings indicate that social influence is strongly linked to prosocial behavior. This implies that socialization and social support for prosocial norms and behavior can exert a powerful influence on the behavior of young people in a Chinese population.

## Introduction

Prosocial behavior is an action primarily intended to benefit others. Sharing and donating resources, comforting others, volunteering for charitable activities, and helping the needy are typical forms of prosocial behavior (1). A wealth of studies shows that prosocial behavior is linked to various aspects of positive youth development (2), including academic success, satisfaction with personal achievement (3), social competence (4), and subjective well-being (3, 5). Interestingly, studies on prosocial development in Chinese adolescents are scarce when compared with the large number of studies on anti-social behavior [e.g., Ref. (6, 7)].

In recent years, there has been growing interest in understanding the development of prosocial behavior as part of positive youth development (8). Prosocial behavior like cooperation is crucial to mutual support and social harmony (9, 10), while helping behavior and volunteering contribute to caregiving in family and social life and instrumental support to others in society (11). Prosocial behavior can also be an important form of social capital for major national or world events like the Olympic Games (12, 13).

Adopting the human ecological perspective (14, 15), this study postulates that forces within adolescents’ micro- and meso-systems (i.e., the individual cognitive and emotional competence of young people and social influence) contribute to the development of prosocial behavior. In particular, many studies supported that moral reasoning and empathy are the key individual competencies that contribute to prosocial behavior. Longitudinal studies had shown that prosocial behavior increased gradually over adolescence, and that the development of prosocial behavior was closely linked to the development of moral reasoning, empathy, and perspective talking. A meta-analysis conducted by Underwood and Moore (16, 17) suggested that moral reasoning and perspective taking (often regarded as the cognitive dimension of empathy) were linked to the development of prosocial behavior.

Empathy and moral reasoning are two individual competences of young people hypothesized to be closely related to prosocial behavior (18, 19). Empathy is generally conceptualized as the ability to examine another person’s perception, feelings, and experience without making judgment, and to communicate one’s understanding concisely to that person (20, 21). Empathy can be dispositional or learned (22), and it involves the interaction of emotional and cognitive processes in understanding people’s feeling, thinking, and experience (23, 24). Empathy has been widely regarded as the foundation of prosocial and altruistic behavior (25), and young people with higher empathy are expected, and found, to be more prosocial (25–27).

Moral reasoning is often regarded to make an impact on the development of prosocial behavior. Based on Piaget’s theory of moral development, Kohlberg (28) proposed six stages of moral development and defined moral reasoning as judgments about right and wrong. Kohlberg defined a subject’s level of moral reasoning from the reasoning used to defend his or her position when faced with a moral dilemma. The stages of moral reasoning indicated an increasing level of moral development, and a higher level of reasoning is associated with more prosocial behavior.

Based on the work of Kohlberg (28), Eisenberg and associates (26, 29) further conducted a series of studies, which showed that moral reasoning becomes increasingly sophisticated from childhood to adolescence, and reaches an empathic orientation stage in which individuals often express sympathetic concern for others. These studies verified that a higher level of moral reasoning is also associated with more prosocial behavior in adolescence.

Young people live in a microsystem in which they may adopt prosocial behavior because of influence from parents, school, or peers (30). It is likely that peer influence has a greater impact on the prosocial and deviant behavior (31) of young people than role modeling by parents (32, 33) or school recognition and teacher support/encouragement of prosocial behavior (6, 7, 10, 34).

The present study aims to address several gaps in the extant research. First, it addresses the scarcity of research on the prosocial development of Chinese populations. Traditional Chinese culture emphasizes the teaching of prosocial norms and modeling of prosocial behavior by parents (35–37). Chinese children are expected to learn to love their family, respect seniors and teachers, build harmonious relationships, and to care for the needy (38). However, there are also increasing complaints about the lack of manners, inconsiderate behaviors, and self-centeredness of Chinese people around the world (39). It would be interesting to examine if prosocial behavior in Chinese populations are different from other cultures.

Second, this study uses the human ecological model to examine how individual competence and social influence affect prosocial behavior. Most previous studies examine the impact of either individual competence or social influence on prosocial development. Few studies have compared the relative importance of individual competence and social influence on prosocial behavior. It is hypothesized that, in a collectivist culture like the Chinese, social influence can have a more significant impact on prosocial behavior than individual choice (9, 27, 37, 39). It is perhaps more important to adhere to social norms and moral expectations for helping and harmonious relationships than to be express one’s compassion and empathy through prosocial acts.

Third, this study recruits a sample of adolescents who participate in volunteering or social service regularly – a prosocial sample. This addresses the methodological issue of low frequency and highly skewed distribution of prosocial behavior in adolescent samples, which is pervasive in studies of prosocial behavior (6). Using a prosocial sample in this study, we can expect a certain level of prosocial behavior in the respondents and it is more feasible to identify the predictors by regression analyses.

Finally, previous local studies show that females generally engage in more prosocial activities than males. Some authors have suggested that adolescent females are more prosocial than males because they have stronger prosocial values (40). There may also be gender differences in the predictors of prosocial behavior, on top of a gender difference in prosocial behavior.

The objective of this study is to identify the predictors of helping intention and prosocial behavior from a number of variables indicating individual psychosocial competence and aspects of social influence. The individual competence variables of empathy and prosocial reasoning, and the social variables of peer, school, and parental influence, were hypothesized to be good predictors of helping intention and prosocial behavior. A secondary objective of this study is to examine if there is a gender difference in predictors of helping intention and prosocial behavior. Ethical approval in conducting this study was granted from University.

## Materials and Methods

### Participants

A convenience sample of secondary students with prosocial characteristics was recruited through a social service and a volunteering organization. The participants fulfilled several criteria (1) aged between 12 and 16 years old, (2) full-time high school student, (3) ethnic Chinese and able to read and understand written Chinese, and (4) participation in at least one volunteering activity regularly (at least biweekly) outside school hours.

A total of 650 questionnaires were distributed and 580 (89%) valid questionnaires were returned. Among the participants, there were 172 (29.5%) males and 408 females (70.5%). The mean age was 14.11 years (SD = 1.22). The majority (61.4%) of participants were junior secondary students and the rest (38.6%) were senior secondary students. Most of the participants regarded their own conduct as excellent (21%), good (51%), or fine (25.3%).

Most of the participants either had one sibling (53.4%) or were the only child (23.6%) in their family, and their parents were married and living together (83.8%). The median household size was four. The median education level of both father and mother was senior high school. Most participants perceived their family life as satisfactory (41.2%) or very satisfactory (28%), and their school life as satisfactory (48.6%) or very satisfactory (24.2%).

### Instruments

The research questionnaire comprised 142 items and incorporated a number of standardized instruments to measure the key variables.

#### Prosocial Behavior

The adolescent behavior questionnaire (ABQ) asks respondents to use a 7-point Likert scale to report on the frequency of altruistic, normative (prosocial), and anti-social acts engaged in at home, at school, and toward peers and strangers in the past year (41). We adopted the prosocial act subscale of the ABQ (19 items), which measures their normative acts (12 items) and altruistic acts (7 items) at home and school. The ABQ had good internal consistency (Cronbach’s α ranged from 0.80 to 0.90), and the ABQ scores correlate positively with measures of altruistic orientation (42, 43).

#### Helping Intention

The adolescent spontaneous helping (AHM) measure was used to measure helping intention, which is defined as the “anticipated future likelihood of engaging in various helping acts” with three groups of people: friends, non-friends, and strangers (44). Respondents were asked to rate, on a 7-point Likert scale, the likelihood that they would provide help in different scenarios. This measured the “helping intention” of adolescents, which is considered the secondary indicator of prosocial behavior in this study. The 24-item Chinese version was validated on 2012. The internal consistency of the AHM subscales and the total score ranged from 0.74 to 0.93 (Cronbach’s α), while the test–retest reliability ranged from 0.75 to 0.88 (ICCs). The reliability estimates are regarded as ranging from “acceptable” to “satisfactory.”

#### Prosocial Reasoning

A translated and modified version of the prosocial reasoning objective measures (PROM) (45), the Chinese prosocial reasoning objective measures (C-PROM) (9) was used to assess the moral reasoning underlying prosocial behavior. The stages of moral reasoning indicated an increasing level of moral development and associated with more prosocial behavior. Therefore, this is directly related to prosocial behavior. C-PROM presented five ethical dilemmas to the respondents and asked them to rate the importance of the stated reasons when they offer their help as stated in these five different scenarios. The C-PROM measured how far respondents adopted five types of moral reasoning (i.e., hedonistic, approval-oriented, needs-oriented, stereotypic, and internalized reasoning), representing their level of moral development. The internal consistency of the C-PROM subscales and the weighted total ranged from 0.74 to 0.93 (Cronbach’s α), while the test–retest reliability ranged from 0.75 to 0.88 (ICCs) (9).

#### Empathy

The Chinese interpersonal reactivity index (C-IRI) is a 21-item self-reported questionnaire measuring empathy-related responses and it has three subscales of personal distress, empathy, and fantasy scale (46). We would use the empathy subscale to measure empathy in this study. Participants were asked to indicate the degree to which each item described them using a 5-point Likert-type scale, which ranged from 0 (“does not describe me well”) to 4 (“describes me very well”). The empathy subscale of C-IRI demonstrated good internal consistency (Cronbach’s α = 0.68) and test–retest reliability (Pearson’s *r * = 0.68) in use with Chinese adolescent samples (46).

#### Peer Influence

The peer interaction questionnaire (PIQ) was used to measure the adolescents’ perception of the influence of their best friend on their prosocial and delinquent behavior (47). In this study, participants were asked to estimate, on a 4-point scale in responding to 6 items, the frequency of prosocial acts performed by their best friend in the past year. The PIQ demonstrated acceptable internal consistency reliability (Cronbach’s α = 0.70) in a previous local study (43).

#### School Influence

Three subscales of the Chinese positive youth development scale (CPYDS) were used to measure school influence on prosocial behavior (48). The 14 items subscales include 4 items on the recognition of positive behavior, 5 items on prosocial involvement, and 5 items on prosocial norms. The findings show that these subscales possess acceptable internal consistency. Cronbach’s α was 0.78 for the “Prosocial Norms” subscale, 0.75 for the “Prosocial Involvement” subscale, and 0.91 for the “Recognition of Positive Behavior” subscale (Cronbach’s α = 0.91) (48).

#### Parental Influence

The Chinese parent helping measure (PHM) was translated and adapted from the Modified Parent Helping Measure (44) used to study parental influence on prosocial behavior. Participants were asked to indicate the likelihood of their parents offering helps to friends and family, and to strangers. The scale has satisfactory internal consistency (Cronbach’s α = 0.86). The 16-item Chinese version was validated on 2012. The internal consistency of the PHM subscales and the total score ranged from 0.74 to 0.91 (Cronbach’s α), while the test–retest reliability ranged from 0.75 to 0.88 (ICCs). The reliability estimates are regarded as ranging from “acceptable” to “satisfactory.”

In the last section of the questionnaire, the participants provided information on socio-demographic background, the type(s) of prosocial behavior they had participated in or had been participating in, and a reflection on their experience of participating in prosocial activities in the past 2 years.

### Procedures

All potential participants were given a briefing about the purpose and information about the study by research staff. All subjects participated on a voluntary basis and a consent form to participate in the study was signed by the students or their parents/guardians. The participants completed the questionnaire under the supervision of the researcher and their class teacher, and most participants completed it within 20 min.

## Results

### Prosocial behavior and helping intention

In the past 2 years, the participants had regularly engaged in one (36.2%) or two (35.2%) volunteering activities, and 28.6% had participated in three or more activities. A large proportion (64.9%) of the participants perceived their experience of volunteering as positive. Descriptive statistics for helping intention, prosocial behavior, individual competence factors, and social influence factors are shown in Table 1.

**Table 1:** **Summary statistics of prosocial behavior and the hypothesized individual and social predictors across gender**.

Variables	Subscales	Total (*N* = 578)		Male (*N* = 171)		Female (*N* = 407)		*t*
		*M*	SD	*M*	SD	*M*	SD	
Prosocial measures	Prosocial behavior	2.22	0.92	2.12	0.97	2.26	0.90	− 1.67
	Helping intention	8.75	3.55	7.97	3.98	9.08	3.32	− 3.42***
Individual competence	Empathy	4.70	0.93	4.86	1.04	4.63	0.88	2.61*
	Prosocial reasoning	1.86	0.06	1.86	0.06	1.86	0.05	0.41
Social influence	Peer influence	1.53	0.55	1.47	0.56	1.55	0.55	− 1.68
	Parental influence	5.84	2.60	5.43	2.92	6.00	2.45	− 2.34*
	School influence	11.15	1.77	10.86	2.11	11.28	1.59	− 2.57*

*p  < 0.05; ***p  < 0.001.

### Gender differences

Female participants had significantly higher helping intention than males (*t* = 3.42, *p* < 0.001), but there were no gender differences in prosocial behavior (*p* > 0.05). Male participants had higher empathy scores (*t* = 2.61, *p * < 0.05). Females perceived their parents as having a higher helping intention than males (*t* = 2.34, *p* < 0.05), and perceived their school as offering more recognition of prosocial behavior than males (*t* = 2.57, *p* < 0.05). However, there was no significant difference in prosocial reasoning and peer influence between female and male subjects (*p* > 0.05) (Table 1).

### Correlates of prosocial behavior and helping intention

The correlation (*r*) between helping intention and prosocial behavior was 0.33 (*p* < 0.001). Age had low and significant positive correlations with helping intention (*r* = 0.14, *p* < 0.01) and peers’ prosocial behavior (peer influence) (*r* = 0.13, *p* < 0.01), but not with prosocial behavior or any of the other predictors. Among the two individual competence variables, empathy had low and significant correlation with prosocial behavior (*r * = 0.23, *p* < 0.01) and with helping intention (*r * = 0.18, *p* < 0.01), while prosocial reasoning did not correlate significantly with either prosocial behavior or helping intention (Table 2). The low but significant correlation between empathy and prosocial reasoning continued when the correlation was calculated separately for female (*r* = 0.18, *p* < 0.01) and male (*r* = 0.16, *p* < 0.01) samples, and there was no significant difference in the magnitude of correlations for males and females.

**Table 2:** **Correlations between potential predictor variables and prosocial behavior and helping intention**.

Correlates	Prosocial behavior	Helping intention
**Socio-demographic variables**
Age	0.04	0.15**
Number of members in household	0.04	0.08*
Number of siblings	0.05	0.01
Father education level	0.04	−0.01
Mother education level	0.08	0.05
Satisfaction with school life	0.16**	0.10*
Satisfaction with family life	0.08	0.05
Satisfaction with volunteering experience	0.25**	0.24**
**Predictors of prosocial measures**
Empathy	0.23**	0.18**
Prosocial reasoning	0.00	0.05
Peer influence	0.43**	0.19**
Parental influence	0.30**	0.69**
School influence	0.38**	0.19**

*p  < 0.05; **p  < 0.01.

All three social influence factors had significant correlations with prosocial behavior. Prosocial behavior had significant correlations with peer influence (*r* = 0.42, *p* < 0.01), school influence (*r* = 0.37, *p* < 0.01), and parental influence (*r* = 0.29, *p* < 0.01). The relationship remained significant when the correlations were calculated separately for males and females. In comparing the correlation between cognitive factors, social factors, and prosocial behavior, it is important to note that there was a significant difference in the magnitude of correlation between parental influence and prosocial behavior across gender (*z* = 2.5, *p* < 0.05), but not for the peer or school influence variables.

### Predictors of helping intention

A regression analysis was conducted to predict the helping intention of adolescents with the adolescent helping measure as dependent variable and parameters covering cognitive factors like empathy and prosocial moral reasoning, and social factors like parental helping, peer helping, and school influence.

The regression model was able to predict a significant proportion of variance in helping intention (*R*^2^ = 0.48) (Table 3). Peer influence and parental influence contributed significantly to the regression model (β = 0.29 and 0.18, respectively), while empathy was significant in predicting helping intention among the two individual competence variables (β = 0.07).

**Table 3:** **Prediction of helping intention from individual competence and social influence factors (*N* = 549)**.

Predictor variables	*B*	SE	β	*t*
**Individual competence**
Empathy	0.25	0.12	0.07	2.06*
Prosocial moral reasoning	1.55	1.98	0.02	0.78
**Social factors**
Peer influence	0.68	0.21	0.11	3.26***
School influence	0.00	0.07	0.00	0.01
Parental influence	0.87	0.04	0.65	20.34***

*p  < 0.05; ***p  < 0.001; *R*^2^ = 0.48, adjusted *R*^2^ = 0.47.

The regression model was able to predict a significant proportion of variance in helping intention for both male and female participants $\left(R_{\text{males}}^{\text{2}}=0.61,\;R_{\text{females}}^{\text{2}}=0.42\right)$ (Table 4). For females, peer influence (β = 0.11, *p* < 0.01) and parental influence (β = 0.61, *p* < 0.001) contributed significantly to the regression model. For males, peer (β = 0.11, *p * < 0.01) and parental influence (β = 0.72, *p* < 0.05) were the most important predictors.

**Table 4:** **Prediction of helping intention from individual competence and social influence factors for males and females**.

Predictor variables	Males (*N* = 172)				Females (*N* = 408)			
	*B*	SE	β	*t*	*B*	SE	β	*t*
**Individual competence**								
Empathy	0.31	0.22	0.08	1.43	0.25	0.15	0.07	1.67
Prosocial reasoning	3.89	3.59	0.06	1.12	1.16	2.41	0.02	0.48
**Social factors**
Peer influence	0.74	0.38	0.11	1.95*	0.64	0.25	0.11	2.57**
School influence	0.02	0.10	0.01	0.19	0.03	0.09	0.01	0.31
Parental influence	0.97	0.08	0.72	12.01*	0.81	0.05	0.61	15.24***

**p  < 0.01; ***p  < 0.001; $R_{\text{males}}^{\text{2}}=0.61,\;R_{\text{females}}^{\text{2}}=0.42$.

### Predictors of prosocial behavior

When the individual competence and social influence variables were used to predict prosocial behavior, the regression model was able to predict a significant proportion of variance in prosocial behavior (*R*^2^ = 0.28) (Table 5). The three social influence variables, peer (β = 0.18, *p* < 0.001), school (β = 0.23, *p* < 0.001), and parental (β = 0.18, *p * < 0.001), contributed significantly to the regression model, while the coefficients of empathy and moral reasoning in individual competence variables were not significant in predicting prosocial behavior (*p * > 0.05).

**Table 5:** **Prediction of prosocial behavior from individual competence and social influence factors (*N* = 580)**.

Predictor variables	*B*	SE	β	*t*
**Individual competence**
Empathy	0.07	0.04	0.07	1.75
Prosocial moral reasoning	−0.36	0.62	0.02	−0.58
**Social influence**
Peers influence	0.49	0.65	0.18	4.81***
School influence	0.12	0.02	0.23	7.55***
Parental influence	0.07	0.02	0.18	4.81***

***p  < 0.001; *R*^2^ = 0.28, Adjusted *R*^2^ = 0.27.

For male participants, the regression model was able to predict a significant proportion of variance in prosocial behavior (*F* = 15.73, *p* < 0.00, *R*^2^ = 0.35) (Table 6). Peer influence, school influence, and parental influence contributed significantly to the regression model (β = 0.22, 0.24, and 0.37, respectively), while the coefficients of the individual competence variables empathy and moral reasoning were not significant in predicting prosocial behavior (*p * > 0.05).

**Table 6:** **Prediction of prosocial behavior from individual competence and social influence factors for males and females**.

Predictor variables	Male (*N* = 172)				Female (*N* = 408)			
	*B*	*SE*	β	*t*	*B*	*SE*	β	*t*
**Individual competence**								
Empathy	0.00	0.07	0.00	0.02	0.08	0.05	0.08	1.77
Prosocial reasoning	−0.05	1.12	0.00	−0.04	−0.48	0.75	−0.03	− 0.64
**Social influence**
Peer influence	0.38	0.12	0.22	3.17**	0.54	0.08	0.33	6.94***
School influence	0.11	0.03	0.24	3.29**	0.13	0.03	0.22	4.52***
Parental influence	0.12	0.02	0.37	5.07***	0.04	0.02	0.11	2.33**

**p  < 0.01; ***p  < 0.001; $R_{\text{males}}^{\text{2}}=0.35,\;R_{\text{females}}^{\text{2}}=0.26.$

For female participants, the regression model was able to predict a significant proportion of variance in prosocial behavior (*F * = 27.90, *p* < 0.00, *R*^2^ = 0.26) (Table 6). Again, peer, school, and parental influence contributed significantly to the regression model (β = 0.33, 0.22, and 0.11, respectively), while the coefficients of the individual competence variables empathy and moral reasoning were not significant in predicting prosocial behavior (*p * > 0.05).

## Discussion

### Predictors of helping intention and prosocial behavior

In this study, researchers found that there was no major issues with multi-collinearity as the figures of VIF of all predictors were <3. Moreover, the results show that social predictors are more important predictors of both prosocial behavior (primary outcome) and helping intention (secondary outcome) than individual predictors. Neither empathy nor prosocial moral reasoning were good predictors of helping intention, and this result runs counter to many studies in other countries that find a strong link between moral reasoning and prosocial behavior (34). It is hard to find similar studies comparing individual and social influence predictors of prosocial behavior and this study implies that social influence could exert much more influence on prosocial behavior than individual prosocial orientation. The results imply that the Chinese adolescent sample in this study could find it very hard to be prosocial when their peers, family, or community do not see the importance of prosocial behavior or are not used to practicing such behavior.

It is noted that the percentage of variance explained in the prediction of helping intention is significantly higher than that of prosocial behavior. This means that the social influence predictors can explain more of the variance in attitude toward helping (helping intention) than prosocial behavior. It also implies that there is probably a gap between willingness to help and the actual implementation of helping or prosocial behavior. This is generally consistent with meta-analyses showing a low but significant correlation of 0.38 between attitude and behavior (49). The execution of prosocial behavior could be reliant on not only just predictors indicative of individual competence or social influence but also factors like the cost or risks to self of helping, or prior experience and reward linked to helping (50).

The present study underscored that social influence from parents, peers, and school play a very important role in the prosocial development of adolescents, which may be explained by cultural norms and socialization practices among Hong Kong Chinese. First, Chinese parenting puts great emphasis on modeling by parents (38) and school socialization (51). Parents in particular are expected to set a good example for children. Second, Chinese individuals tend to have a strong inclination to align and compare themselves with peers, and thus peer influence can have great impact on the development of prosocial and anti-social behavior (52). It is likely that the prosocial sample had prosocial peers who contribute to their helping intention and prosocial behavior. Third, being prosocial can be a way to save face (53). Chinese people give great consideration to what others do in deciding what they themselves should do. It would be shameful to be accused of not being prosocial or helpful, and this could motivate people to act more responsively to the needs of others. Furthermore, culture and the media are likely to have an impact on the valorization of prosocial behavior (1), and further research should focus on the role of the media in prosocial behavior development.

For both prosocial behavior and helping intention, parental influence is the most important predictor, followed by peer and then school influence. These findings are in different from literatures, which showing the great impact of peer influence on adolescent development (31). Parental influence and modeling have long been found to be positively related to the early prosocial development of children and adolescents, especially in children and early adolescents (54, 55).

Peers may exert their influence on adolescents’ prosocial behavior via direct interpersonal influence or modeling (1). It is worth noting that peer influence among females is more strongly related to prosocial outcomes than among males. This implies that peer influence could have a greater impact on females than on males, which could be explained by the stronger social networks and support found among adolescent girls (56). Moreover, when children reach adolescence, they tend to listen to their peers more than their parents (57, 58), and peers gradually become a more significant social resource than parents (59–61).

School influence is another important predictor of prosocial behavior after peer influence. The results reinforce previous findings that positive school influence is related to prosocial behavior and socio-emotional adjustment (62). A positive school culture that emphasizes connectedness and cooperation can facilitate positive peer relations (1, 63), protect adolescents from experiencing emotional and behavioral problems (64), and create a context vital to the development of self-esteem (65).

Moreover, this study did not try to conduct structural equation modeling (SEM), as it is hard to put up a well-justified hypothesized model for testing at this time. This is one of the very few studies to examine both individual and social predictors of prosocial behavior, as most previous studies use individual cognitive or emotional characteristics as predictors. Therefore, it is not mature to use SEM at this stage, but it could be a recommendation for further study.

### Study limitations

There are several limitations in this study. First, although the predictors contributed significantly to the prediction of helping intention and prosocial behavior, the percentage of variance explained in prediction (*R*^2^) was 0.48 for helping intention and 0.28 for prosocial behavior. This means that there are other predictors that were not included in this study, especially for the prediction of prosocial behavior. These variables may include prior experience of helping, socio-cognitive abilities, sympathetic tendencies of individuals, as well as contextual factors (e.g., the cost of helping) (26, 66, 67). However, the inclusion of more predictors would lengthen the research questionnaire, making it less acceptable to participants.

Second, female participants represented 70% of this prosocial sample group. In exploring gender differences, female participants presented higher helping intention than males, but both groups presented similar levels of prosocial behavior. Females also perceived their parents as more prosocial and their school as more supportive of prosocial behavior than males. The overall conclusion of the study could be affected by these gender differences. However, when we conducted regression analyses on the male and female samples, the significant predictors remained the same, although there were some differences in the variance explained.

Third, the recruitment of a prosocial sample in this study addresses the highly skewed distribution and low frequency of prosocial behaviors in adolescent populations, which enables the identification of predictors using regression analyses. It should be noted that the study results may thus apply mainly to young people who have a history of volunteering or are used to providing help to others. This group of participants may have a tendency toward social desirability in responding to questionnaires. However, we did not find significant differences in empathy and prosocial reasoning between this sample and two other, larger samples in our previous studies (9, 27), although the current sample has significantly more prosocial behavior. Thus, we do not consider social desirability to be a major issue affecting the validity of the results.

Fourth, it should be noted that the variables indicating peer, family, and school influence are indicated by variables measuring how participants perceived the prosocial behavior of their parents and peers, and how they perceived their schools as supportive of volunteering and helping behavior. These are not exactly social influence variables, but are measured to reflect the degree of social influence.

Last, it was observed that some participants found the Chinese PROM rather demanding to complete. It requires participants to understand five ethical scenarios and think through the different reasons for giving or not giving help. This section of the research questionnaire tends to demand more time and attention than other sections, and some participants may tend to hurry through it rather than reflecting deeply on their responses. The ethical dilemma format has been used as a standardized format measuring moral or prosocial reasoning, but it is relatively more challenging to administer to a large group.

## Conclusion

This is a pioneering study comparing individual competence and social influence predictors of prosocial behavior as the primary outcome, and predicting helping intention as the secondary outcome. Social influence predictors have been identified as more important in predicting both prosocial behavior and helping intention than individual competence predictors. Parental influence, peer influence, and school influence were noted to be significant predictors in social influence. In testing individual competences, neither empathy nor prosocial moral reasoning was a good predictor of helping intention and prosocial behavior in Hong Kong Chinese adolescents. These findings differ from previous findings in foreign countries, which identify a strong link between empathy and moral reasoning and prosocial behavior. The percentage of variance explained in the prediction of helping intention is significantly higher than the prediction of prosocial behavior. The results of this study provide an important reference for parents and educational and social services who want to cultivate prosocial behavior among young people.

The findings of this study needed to be interpreted with the following considerations. First, use of prosocial sample was intended to address social desirability in the methodology of this study. However, it might limit generalization of results to general population. Second, female subjects predominated in studied population; therefore, gender difference could not be fully examined. Third, measure of social influence variables are all based on adolescents’ subjective rating to their friend, their subjective reflection of school culture and their own estimation of parental helping behavior. It may not totally reflect the social influence.

To conclude, the prosocial behavior and helping intention of adolescents were studied broadly, and the inter-relationships among these variables became clearer as specific patterns among them were emerged. Based on this pioneer study, further research is necessary, but the examination of prosocial behavior of adolescent provided here could serve as a strong base for future research.

## Conflict of Interest Statement

The authors declare that the research was conducted in the absence of any commercial or financial relationships that could be construed as a potential conflict of interest.
